# Dexmethylphenidate-Induced Rhabdomyolysis by Interaction With Aromatase Inhibitor

**DOI:** 10.7759/cureus.27988

**Published:** 2022-08-14

**Authors:** Derrick Huang, Shane Dluzneski, Michael Hughes, Samyr Elbadri, Latha Ganti

**Affiliations:** 1 Emergency Medicine, University of Central Florida College of Medicine, Ocala, USA; 2 Emergency Medicine, HCA Florida Ocala Hospital, Ocala, USA; 3 Emergency Medicine, Envision Physician Services, Plantation, USA; 4 Emergency Medicine, University of Central Florida College of Medicine, Orlando, USA

**Keywords:** non-traumatic rhabdomyolysis, amphetamine-dextroamphetamine, aromatase inhibitor, attention deficit hyperactivity disorder (adhd), pituitary hormone replacement, letrozole, dexmethylphenidate, growth hormone replacement therapy

## Abstract

Rhabdomyolysis secondary to prescription drug-drug interactions can be an overlooked life-threatening emergency. Amphetamines and similar substances have been associated with muscle lysis secondary to increased sympathetic activity that can cause myotoxicity, hyperthermia, and increased muscular activity. Anabolic steroids may also be a predisposing factor in developing rhabdomyolysis. A high index of suspicion for drug-induced rhabdomyolysis in a patient presenting with atraumatic extremity pain can facilitate rapid diagnosis and treatment. We present a case of drug-induced rhabdomyolysis likely secondary to a previously unreported medication interaction.

## Introduction

Rhabdomyolysis is a relatively common diagnosis with approximately 25,000 pediatric and adult cases reported annually in the United States [1]. This pathology is typically defined by the lysis of skeletal muscle tissue that results in the release of toxic intracellular material into the systemic circulation secondary to etiologies such as infection, trauma, drugs, and inherited disorders [1,2]. A definitive diagnosis requires an elevation of creatinine kinase (CK) greater than five times the upper limit of normal according to age, gender, and race [1-3]. The release of intracellular material into the systemic circulation can lead to electrolyte disturbances, hypovolemia, metabolic acidosis, coagulation defects, and acute renal failure due to myoglobinuric intratubular cast formation [1,2].

In the emergency department (ED), the primary focus in atraumatic extremity muscle pain is rapid diagnosis and treatment of life-threatening pathologies. This assessment can be complicated by a wide array of possible etiologies, including infection, autoimmune and endocrinologic pathologies, and electrolyte abnormalities. In particular, a thorough medication history may facilitate diagnosis and expeditious treatment of etiologies that include neuroleptic malignant syndrome, serotonin syndrome, and drug-drug interactions [4]. Here, we present a case of rhabdomyolysis likely secondary to a previously unreported prescription medication interaction.

## Case presentation

A 14-year-old male with a past medical history of attention-deficit hyperactivity disorder (ADHD) and pituitary dwarfism presented to the ED for bilateral upper extremity pain. He stated that his arms have had a worsening burning sensation beginning approximately three days prior to arrival. He denied prior episodes with similar symptoms, recent engagement in strenuous physical activity, and non-prescription supplement use. He denied fevers, nasal congestion, coughing, chest pain, nausea, vomiting, abdominal pain, and changes in urine color. On his medication history, the patient started to take growth hormone therapy with somatotropin and letrozole, an aromatase inhibitor, one month ago for his diagnosis of growth hormone deficiency. Of note, he also restarted his ADHD medication, dexmethylphenidate extended release, one day prior to the onset of his arm pain. He was on similar medications in the past and was initially recommended to avoid taking them along with his other medications; however, given that his grades were starting to drop, his endocrinologist agreed he could resume his ADHD medication.

On his initial vitals, he had a blood pressure of 109/63 mmHg, heart rate of 64 beats per minute, temperature of 36.9°C, and respiratory rate of 18 breaths per minute. The patient had a height of 1.57 m (about 5 ft 2 in), a weight of 47.1 kg (about 103.8 lbs), and a BMI of 19.11 kg/m². He was alert, active, and cooperative. His upper extremities were diffusely tender to palpation with a diffusely decreased range of motion due to pain. Visual inspection of his extremities was unremarkable and his strength was intact. His initial labs were remarkable for an initial CK level of 9978 U/L. His blood urea nitrogen, creatinine, and urinalysis were unremarkable. He was immediately given an intravenous crystalloid bolus and started on maintenance fluids. While inpatient, the patient continued to receive aggressive hydration with close monitoring of his CK levels. After his CK levels continued to downtrend with associated clinical improvement, the patient was discharged home with temporary restrictions to physical exertion. His endocrine and ADHD regimens were discontinued until clinical improvement back to baseline for reassessment and possible re-initiation of treatment.

## Discussion

**Figure 1:**
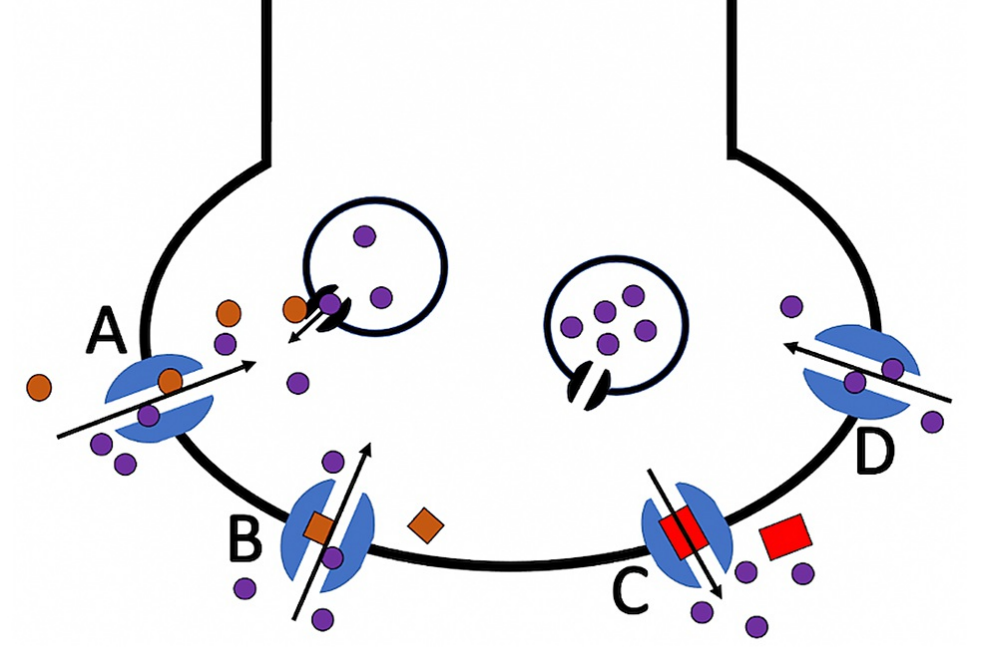
Illustration of a neuronal synapse with monoamine transporters showing different mechanisms of action of amphetamine, methylphenidate, and cocaine Illustration of a neuronal synapse with monoamine transporters showing different mechanisms of action of amphetamine (A), methylphenidate (B), and cocaine (C). No drug (D) is also illustrated for comparison. In (A) and (B), both amphetamine and methylphenidate compete with the monoamine, norepinephrine (purple circles), for entry into the neuron, resulting in increased norepinephrine remaining in the synaptic cleft. Amphetamine also binds to synaptic vesicles inside the neuron, which results in increased monoamine release. In (C), cocaine binds and completely blocks the monoamine transporter.

To our knowledge, we report a case of rhabdomyolysis likely secondary to a previously unreported medication interaction between letrozole, somatotropin, and dexmethylphenidate. In the pediatric population, medication-related rhabdomyolysis is relatively rare, with a rate of less than 5% in a study of cases diagnosed and treated in the ED of a tertiary pediatric hospital over a decade [3]. Although substances like dexmethylphenidate can independently cause delayed rhabdomyolysis, the onset of symptoms after our patient's re-initiation of this drug superimposed on medications that may promote myotrophy lends credence to a drug-drug interaction [5-7]. As the patient was afebrile without signs of infection, did not engage in drug abuse and strenuous activity, and without similar prior episodes to suggest a genetic etiology, the most likely etiology of his rhabdomyolysis was a synergetic interaction between his letrozole, somatotropin, and dexmethylphenidate [5-7]. As described in a prior report of anabolic steroid and cocaine use, the simultaneous use of medications involving anabolic steroids and substances promoting norepinephrine and dopamine release can result in rhabdomyolysis (Figure 1) [6]. In our case, dexmethylphenidate may be pharmacodynamically analogous to amphetamine as well as cocaine via its inhibition of the reuptake of norepinephrine and dopamine into the presynaptic neuron by competitive uptake with monoamines at the dopamine and norepinephrine transporters (Figure 1) [8-10]. This indirectly increases catecholaminergic neurotransmission and sympathetic stimulation, which results in increased muscular activity, hyperthermia, and vasoconstriction that may lead to muscle ischemia [2,11]. Furthermore, letrozole acts by inhibiting the enzyme aromatase, which converts androgens into estrogens, increasing the androgen substrate, testosterone. This well-known androgen has been described as an etiology of rhabdomyolysis via an unknown mechanism associated with a myotrophic state [12].

Trauma and exercise are the most common precipitants of rhabdomyolysis in adults and the frequency of these etiologies is also increasing among teenagers [1]. Athletes and the military are at a higher risk due to the potential of abrupt changes in activity level between periods of activity and rest. Other concomitant risk factors linked to exertional rhabdomyolysis include sickle cell trait, electrolyte derangements, high BMI, eccentric modes of exercise, and environmental elements such as high humidity and altitude [1]. Differing from adults, the most common cause in children in the first decade of life is a viral infection, most commonly reported from influenza, Epstein-Barr virus, and cytomegalovirus. This is followed by inherited pathologies, particularly in the setting of recurrent rhabdomyolysis, such as disorders of lipid and glycogen metabolism, mitochondrial dysfunction, and myopathies, such as carnitine palmitoyltransferase II deficiency and McArdle disease [1,3]. Rhabdomyolysis related to intoxication is more common in adults, with notable agents including alcohol, involving both intoxication and withdrawal, and drugs, such as cocaine, marijuana, heroin, ecstasy, and lysergic acid diethyl-amide, and anabolic steroids [1]. As seen in our case, pediatric patients also deviate significantly from the classic triad of myalgia, weakness, and dark urine. In one large retrospective chart review of pediatric rhabdomyolysis presenting in the ED of a tertiary pediatric hospital, presentation with the full triad was exceedingly rare, occurring in only one case out of 191 studied [3,13].

Prompt treatment of rhabdomyolysis is essential in the prevention of complications resulting from the release of toxic intracellular components. The cytotoxic renal intratubular cast formation due to myoglobin release in rhabdomyolysis can result in an acute kidney injury (AKI), accounting for as many as 7% of all cases of AKI in the United States [14]. As in our case, early recognition and aggressive hydration are essential in the prevention of AKI [3,14]. In adult cases of severe rhabdomyolysis, there may be a theoretical benefit in the alkalinization of urine with sodium bicarbonate to prevent heme-protein precipitation and intratubular pigment cast formation. However, studies supporting this benefit were derived from uncontrolled case series in adults with severe rhabdomyolysis, and there is no data suggesting effectiveness in children [3,14,15]. Patients also require assessment for the presence and development of metabolic abnormalities including hyperkalemia, usually treated medically or potentially with dialysis, and hyperuricemia, typically treated with allopurinol [1,2,14]. Given the potential for the late occurrence of hypercalcemia and the risk of calcium-phosphate precipitation, calcium supplementation for the treatment of hypocalcemia is generally avoided in the setting of rhabdomyolysis [16]. There is no specific additional therapy in patients complicated by an AKI; however, initiation of dialysis may be required to treat volume overload, hyperkalemia, severe acidemia, and uremia [16,17]. When complicated by an AKI, the mortality rate in rhabdomyolysis may be as high as 80%, especially when CK levels reach 100,000 U/L [14]. The risk of AKI in rhabdomyolysis in adults may range from 17% to 35%, whereas in children, this risk may be as low as 5% with expeditious fluid treatment [13,14]. Factors that may predict renal failure include dark urine, initial and peak myoglobin levels, body temperature, and hyperkalemia [18].

## Conclusions

Rhabdomyolysis secondary to prescription drug-drug interactions can be an overlooked life-threatening emergency. In the setting of a patient with atraumatic extremity pain, a thorough medication history that incorporates an understanding of pharmacologic mechanisms can increase the index of suspicion for drug-induced rhabdomyolysis and facilitate rapid diagnosis and treatment. 
